# Local Path Planning of Autonomous Vehicle Based on an Improved Heuristic Bi-RRT Algorithm in Dynamic Obstacle Avoidance Environment

**DOI:** 10.3390/s22207968

**Published:** 2022-10-19

**Authors:** Xiao Zhang, Tong Zhu, Lei Du, Yueqi Hu, Haoxue Liu

**Affiliations:** 1 School of Automobile, Chang’an University, Xi’an 710064, China; 2 College of Transportation Engineering, Chang’an University, Xi’an 710064, China; 3 School of Vehicle Engineering, Xi’an Aeronautical Institute, Xi’an 710077, China

**Keywords:** autonomous vehicle, local path planning, Bi-RRT, path reorganization, path coherence

## Abstract

The existing variants of the rapidly exploring random tree (RRT) cannot be effectively applied in local path planning of the autonomous vehicle and solve the coherence problem of paths between the front and back frames. Thus, an improved heuristic Bi-RRT algorithm is proposed, which is suitable for obstacle avoidance of the vehicle in an unknown dynamic environment. The vehicle constraint considering the driver’s driving habit and the obstacle-free direct connection mode of two random trees are introduced. Multi-sampling biased towards the target state reduces invalid searches, and parent node selection with the comprehensive measurement index accelerates the algorithm’s execution while making the initial path gentle. The adaptive greedy step size, introducing the target direction, expands the node more effectively. Moreover, path reorganization minimizes redundant path points and makes the path’s curvature continuous, and path coherence makes paths between the frames connect smoothly. Simulation analysis clarifies the efficient performance of the proposed algorithm, which can generate the smoothest path within the shortest time compared with the other four algorithms. Furthermore, the experiments on dynamic environments further show that the proposed algorithm can generate a differentiable coherence path, ensuring the ride comfort and stability of the vehicle.

## 1. Introduction

The intelligent transportation system is a real-time, accurate, efficient, and comprehensive transportation management system that plays a role in various directions [1]. It can effectively improve road capacity, reduce traffic accidents, improve transportation efficiency, and alleviate traffic congestion [2,3]. Meanwhile, it can also reduce energy consumption and improve environmental pollution [4,5]. Therefore, it has become the future development direction of the transportation system and has attracted more and more attention from all countries. As a component, the autonomous vehicle plays an essential role in the intelligent transportation system. It consists of an environmental perception layer, a path planning layer, and a path tracking control layer, and the study of path planning has always been a core problem. Commonly, path planning refers to efficiently finding a collision-free and feasible path from a starting point to a target point in a workspace [6,7,8]. In practical usage, the quality of the planned path will directly affect the vehicle’s driving performance, so how to plan a passable path that can be tracked is very important for autonomous vehicles.

Scholars have carried out much research on path planning, and new path-planning algorithms are constantly emerging and developing. In the previous studies, five common categories of path planning algorithms can be found: geometric algorithms [9,10], graph search algorithms [11,12], intelligent bionic algorithms [13,14], the artificial potential field algorithm [15], and sampling-based search algorithms. Sampling-based search algorithms, including the rapidly exploring random tree (RRT) and the probability roadmap, have effectively solved many challenging planning problems, especially in complex environments [16,17]. In path planning, the basic RRT algorithm is widely used for actuators with nonholonomic constraints because of the advantages, including probability completeness, low computational cost, and no need to model search space [18,19,20]. However, the basic RRT only focuses on finding a path, with less regard to the convergence speed, the search efficiency, and the path optimality [21,22,23]. To overcome the basic RRT’s shortcomings, some scholars made many improvements. The biased RRT uses target-biased search to form an extended mode of nonrandom sampling, thus improving planning efficiency [24]. The bidirectional RRT (Bi-RRT) can simultaneously generate two trees from the starting point and the target point to explore the search space, improving the algorithm’s search efficiency [25]. The RRT-connect, a Bi-RRT version fusing a greedy function, generates two trees from the starting point and the target point, respectively, which reduces the search space and accelerates the convergence speed of the algorithm [26]. The RRT* uses new steps, including reselecting the parent node and rewiring the neighboring nodes of the newly inserted node to change search mode, thus generating a path with the optimal or approximate optimal length [27]. These algorithms improve the performance in planning speed and path length, respectively. However, they do not take the steering constraints of the wheels into account, resulting in them not being applied to the path planning of the autonomous vehicle directly.

When local practical environments are partially known or dynamic, supposing that some unknown or dynamic obstacles occupy the pre-generated global path at a certain moment, the autonomous vehicle will collide with the obstacles while tracking the path. Therefore, to avoid dynamic obstacles, the autonomous vehicle needs to regenerate a feasible path in real-time according to the environmental information obtained from its perception module. Park et al. constructed an algorithm combining the A* method and the artificial potential field method to solve online local path planning problems in the campus environment, guaranteeing real-time performance and the shortest path generation [28]. Chen et al. use a two-layered path planning model structure consisting of the modified Bi-RRT based on the steering constraint and a vector field histogram-guided polynomial planning method to plan a safe and smooth path meeting the real-time requirement [29]. Ge et al. utilized the resultant force of the potential field, the separating axis theorem, and the cubic B-spline to improve the Bi-RRT* and take the vehicle constraints into account, resulting in obtaining the smoothest path by taking the shortest time in practice the complicated environment [30]. Qi et al. utilized a modified RRT* to obtain an initial path, regard the state tree structure as prior knowledge, and design an approach to choose the best node among several candidates to regenerate the path quickly, resulting in planning a path avoiding dynamic obstacles [31]. Zou et al. proposed a path-planning algorithm based on RRT and reinforcement learning optimization, which can generate a smooth and steady path in complex and unknown environments without collision with obstacles [32]. Li et al. presented a real-time RRT-based path planning strategy consisting of a pre-processing RRT path planner and a real-time planner, which can modify the path rather than regenerate a path to avoid the unknown moving obstacle [33]. Peng et al. introduced a new way to choose candidate nodes, incremental step size, and the rapidly exploring random vines with a trajectory parameter space to form a kinematically constrained RRT-based path planning algorithm, which can find collision-free and kinematically feasible paths in various environments, such as dense environments and environments with narrow passages [34]. Wen et al. employed environmental knowledge to guide the planning procedure of the optimal RRT* method to propose a heuristic dual sampling domain reduction-based optimal RRT scheme including a layered online path planning framework in accordance with the model predictive control method, which outperforms traditional reduction schemes in terms of improving the execution efficiency of RRT* and is more reliable [35]. Niu et al. proposed a global dynamic path planning method based on an improved A* algorithm and combine it with the dynamic window method to improve the real-time performance of the dynamic obstacle avoidance of the intelligent vehicle [36]. Wu et al. utilized the genetic algorithm to optimize the path length and turning angle to obtain a short and smooth path [37]. The above path planning algorithms have improved the length and smoothness of the path and can be applied in a dynamic environment. However, they seldomly consider the curvature consistency of paths in multiple frames which refers to the fact that there is no sudden change between the path planned in the current frame and the path planned in the previous frame.

Furthermore, path optimization can effectively reduce the control difficulty of autonomous vehicles with nonholonomic constraints. Ge et al. used the cubic B-spline directly to optimize the path, resulting in the planned path with more turns [30]. Lu et al. utilized Dubins curves to generate a path, but the curvature of the generated path is discontinuous [38]. Yang et al. used path pruning to delete unnecessary path nodes without considering the included angles between line segments between path nodes, resulting in excessive curvature of the final planned path [39]. Chen et al. adopted path pruning based on inserted points to solve the initial path without considering the influence of inserted points on the path length, causing the length of the final planned path may not be optimal [29]. Thus, developing a path optimization method that can consider both path pruning and smoothing is necessary.

Therefore, in this article, an improved heuristic Bi-RRT path planning algorithm is proposed to solve local path planning problems of the dynamic environment by considering path length and the continuity of path curvature between frames. The improved heuristic Bi-RRT algorithm has contributed to node sampling, node selection, node extension, the interconnection mode of two trees, path organization, and the coherence of path curvature between frames.

1. The multiple-sampling states plus a guided method biased towards the target point are designed to reduce the blind growth of the random trees, and the node extension mechanism integrating the greedy algorithm, namely the adaptive greedy step size considering the target direction, can effectively accelerate the growth of two random trees.
2. The nearest node selection mechanism considering the kinematic constraint of the vehicle and the target state is put forward to reduce the effect of random sampling on path smoothness and speed up the growth of the random trees.
3. An amplifying vehicle constraint considering the driver’s driving habit is introduced to make the vehicle move more safely, and the obstacle-free direct connection mode of two trees is introduced to further accelerate the execution of the algorithm.
4. A path reorganization process is designed to optimize the initial path to decrease the length of the final planned path to the maximum extent while ensuring path smoothness.
5. A novel path coherence method considering the inter-frame correlation of paths is used to ensure the curvature continuity of the path and make the vehicle be controlled easily and move more steadily.

The article is organized as follows: Section 2 discusses the Bi-RRT path planning algorithm, the simplified vehicle model, and the differences in path planning between front and back frames. The improved heuristic Bi-RRT algorithm is presented in Section 3. Section 4 presents simulation experiments to demonstrate the effectiveness and practicability of the proposed algorithm. Section 5 presents the discussion about its performance, and conclusions are provided thereafter.

## 2. Problem Statements

The basic bi-RRT algorithm is briefly described in this section, and its shortcomings are pointed out. A brief introduction of the vehicle model points out that the turning radius constraint of the vehicle needs to be considered in the path planning process. Furthermore, the influence of the difference between the path planning results of the front and rear frames in the dynamic path planning process on vehicle driving is described.

### 2.1. Basic Bi-RRT

**Figure 1:**
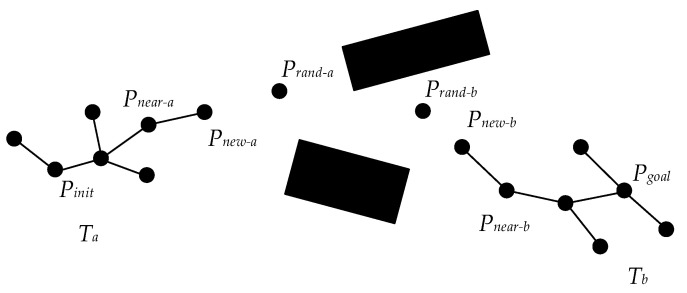
Schematic diagram of basic Bi-RRT expansion.

The Bi-RRT is a variant of the basic RRT, which changes the expansion mode of the algorithm. That is, two random trees are constructed from the initial state and the target state, respectively. In each cycle, a random tree is first expanded to generate a new tree node, and then another random tree also starts to generate a new tree node, making two random trees expand towards each other. The two random trees expand alternately until the nodes of the two random trees meet. The searching schematic diagram of the basic Bi-RRT is shown in Figure 1.

Algorithm 1 shows the basic Bi-RRT algorithm. Once initialized, the basic Bi-RRT algorithm conducts its iterative circle by selecting a random point $P_{rand}$ from the configuration space using the sampling function Random_State ( ) (Line 3). The algorithm then determines a near tree node $P_{near}$ by the function Nearest_Neighbor ( ) and obtains a new tree node $P_{new}$ by the function Extend ( ) (Lines 4–5). If there are no obstacles between $P_{near}$ and $P_{new}$, the new tree node $P_{new}$ is added to the random tree $T_{a}$, and the nearest tree node $P_{nearest}$ from the random tree $T_{b}$ is found by the function Nearest_Neighbor ( ) (Lines 6–8). The iterative circle terminates if the distance between $P_{new}$ and $P_{nearest}$ is less than $l_{threshold}$ (Lines 9–11). Otherwise, $T_{a}$ and $T_{b}$ are swapped, and the procedures mentioned above are executed on the random tree $T_{b}$ again (Line 12). Additionally, then, a path is generated by the function Get_Path T ( ) (Line 16).
**Algorithm 1:**$\mathrm{Build}\;\mathrm{Bi}-\mathrm{RRT}\;(P_{init}$,$P_{goal}$)1: $T_{a}\;(P_{\mathrm{init}})$; $T_{b}\;(P_{goal})$; 2: **while 1 do** 3:  $P_{rand}\leftarrow Random\_State\;(\;)$; 4:  $P_{near}\leftarrow Nearest\_Neighbor\;(P_{rand},T_{a})$; 5:  $P_{new}\leftarrow Extend\;(P_{near},P_{rand})$; 6:  **if** $\;Collision\_Free\;(P_{near},P_{new})$ **then** 7:  $T_{a}.Add\;(P_{new}),\;T_{a}.Add\;(P_{near},P_{new})$ 8:  $P_{nearest}\leftarrow Nearest\_Neighbor\;(P_{new}\;,T_{b})$; 9:  **if** $Dis\tan ce\;(\;P_{new},\;P_{nearest})<l_{threshold}$ **then** 10:    Return T (Ta, Tb) 11:    break 12:    **else** $Swap\;(\;T_{a},T_{b})$ 13:  **end if** 14: **end if** 15: **end while** 16: $Path\leftarrow Get\_Path\;T\;(\;T_{a},\;T_{b})$;

Algorithm 2 outlines the implementation procedure of the function Get_Path T ( ). Once the basic Bi-RRT algorithm completes the construction of two random trees, $T_{a}$ and $T_{b}$, two path point sets, path_a and path_b, are defined, and the last added tree nodes of the two random trees are put into two sets, path_a and path_b, respectively (Lines 1–3). Then, the two random trees are searched reversely according to indexes of parent nodes until their initial points are put into the path point sets, path_a and path_b, respectively (Lines 4–17). Finally, the path point set path_a is reversed and then combined with the path point set path_b to obtain a final path point set path (Lines 18–19).
**Algorithm 2:** Function Get_Path T ( Bi−T)1: Var path_a, path_b; 2: $path\_a.Add\_Node\;(T_{a}.node_{n})$; 3: $path\_b.Add\_Node\;(T_{b}.node_{n})$; 4: **while 1 do** 5: $i\leftarrow Index_{pre\_node}\;(T_{a})$; 6: $path\_a.Back\_Add\_Node\;(T_{a}.node_{i})$; 7:  **if** i=1 **then** 8:   break 9: **end if** 10: **end while** 11. **while 1 do** 12: $j\leftarrow Index_{pre\_node}(T_{b})$; 13: $path\_b.Back\_Add\_Node\;(T_{b}.node_{j})$; 14: **if** j=1 **then** 15:  break 16: **end if** 17: **end while** 18: path_a←reverse (path_a); 19: path←path_a∪path_b; 20: Return path


The basic Bi-RRT algorithm simultaneously generates two random trees from the starting and target points and expands them in opposite directions, accelerating the convergence speed of the algorithm. However, the expansion mode of tree nodes still lacks directivity, the connective mode of two trees can be further improved, and the generated path is difficult to be directly tracked by the vehicle.

### 2.2. Vehicle Kinematical Model

**Figure 2:**
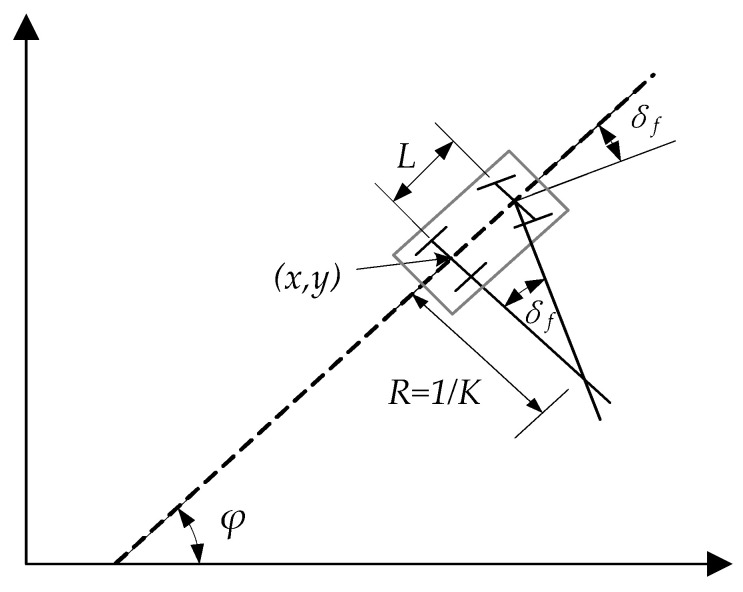
Schematic of vehicle kinematic model.

Since the Bi-RRT is an incremental path planning algorithm, the kinematic vehicle model can be used to limit the expansion process of tree nodes to ensure the feasibility of the path. That is, the nonholonomic constraint of the vehicle should be considered when increasing new tree nodes. Because the sophisticated kinematic model is seldom available, a simplified theoretical motion model is provided, as shown in Figure 2. Assuming that the vehicle does not slip laterally, and the rear wheels do not steer, the vehicle kinematic model is expressed by Equation (1). Furthermore, the steering radius of the vehicle can be expressed by Equation (2).

(1) $$\left\{\begin{array}{l} \dot{x}=v\cos\varphi \\ \dot{y}=v\sin\varphi \\ \dot{\varphi }=\frac{v\tan\delta_{f}}{L} \end{array}\right.$$

(2) $$\left|k\right|\leq k_{\max}=\frac{1}{L}\tan\delta_{f_{\max}}=\frac{1}{R_{\min}}$$

where $\left(x,y\right)$ represents the coordinate of the vehicle gravity center in the coordinate reference frame, v is the longitudinal speed of the vehicle, φ is the included angle between the vehicle main axis and the X axis, $\delta_{f}$ is the steering angle of the front wheels and $\left|\delta_{f}\right|\leq \delta_{f_{\max}}$, L is the wheelbase of the vehicle, k and R are the turning curvature and steering radius of the vehicle, respectively.

In order to consider the feasibility of newly generated path segments, the minimum steering radius constraint should be taken into account during the node extension procedure of Bi-RRT.

### 2.3. Path Planning Difference between Previous and Subsequent Frames

**Figure 3:**
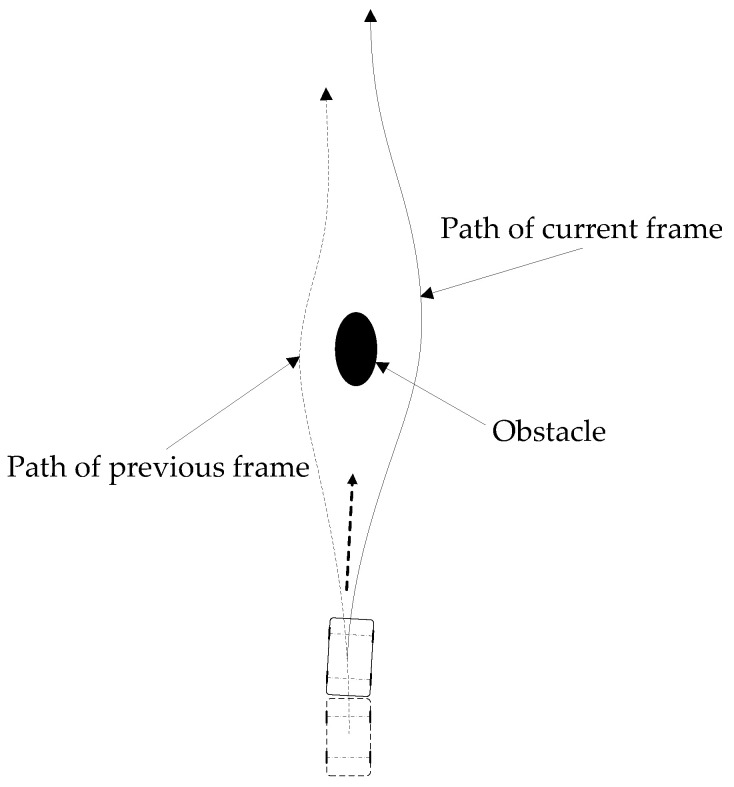
Path difference between current and previous frames.

Because the length and smoothness of the path are calculated based on the environmental information collected in a certain frame, only considering the length and smoothness of the path cannot guarantee the steady driving of the autonomous vehicle. Suppose the path planned in the current frame deviates too far from the previous one. In that case, the driving stability of the autonomous vehicle will decline and even collide with the obstacle vehicle. It can be seen from Figure 3 that the path of the previous frame of the autonomous vehicle is on the left side of the obstacle, whereas the path of the current frame is on the right side of the obstacle. Because of the inconsistency of the path of the previous and subsequent frames, the autonomous vehicle may not avoid the obstacle and has the risk of collision with the obstacle. The dotted arrow may represent the actual driving direction of the autonomous vehicle.

Consequently, to prevent the difference in the paths generated by the previous and current frames of two adjacent planning cycles from influencing vehicle driving stability, it is necessary to consider the path information of the previous frame when planning the path in the current frame.

## 3. Improved Heuristic Bi-RRT Algorithm

**Figure 4:**
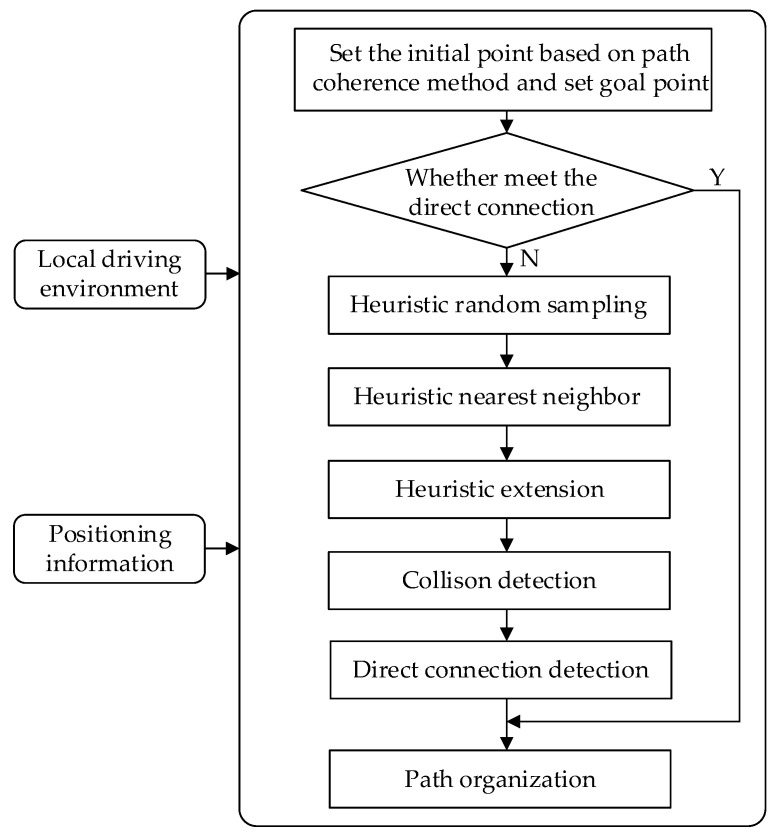
Model structure of the improved heuristic Bi-RRT.

Based on the above analysis, this section proposes an improved heuristic Bi-RRT algorithm for path planning in a dynamic obstacle avoidance environment. Figure 4 illustrates the model structure of the improved heuristic Bi-RRT. The input of the proposed model is a local driving environment and positioning information. The proposed algorithm model based on heuristic random sampling, heuristic nearest neighbor, heuristic extension, collision detection, direct connection detection, and path organization quickly generates a differentiable and collision-free path. Algorithm 3 shows the specific steps of the improved heuristic Bi-RRT algorithm.

The improved heuristic Bi-RRT algorithm obtains an initial point by the function Current_Root ( ) and initializes two random trees $T_{a}$ and $T_{b}$ in the same manner as they are in the basic Bi-RRT (Lines 1–2). Once the two random trees cannot be directly interconnected, the improved heuristic Bi-RRT begins its iterative processing by picking a random point in the feasible domain space through a sampling function Heuristic_Random_Sampling ( ) (Lines 4–5). Then, the parent node $P_{near}$ from tree $T_{a}$ is found by the function Heuristic_Nearest_Neighbor ( ), and the new tree node $P_{new}$ is generated by the function Heuristic_Extend ( ) (Lines 6–7). If there are no obstacles between $P_{near}$ and $P_{new}$, the new tree node $P_{new}$ is added to the random tree $T_{a}$, and the nearest node $P_{nearest}$ from tree $T_{b}$ is found (Lines 8–11). Subsequently, the iterative processing ends if there are no obstacles between $P_{nearest}$ and $P_{new}$, namely $P_{judge2}$ and  respectively (Lines 13–15). Otherwise, random trees $T_{b}$ and $T_{b}$ are swapped, and the procedures mentioned above are executed on the other random tree $T_{b}$ again (Line 16). Afterward, an initial path is generated by the function Get_Path T ( ) (Line 20). Path organization, including path node reconnection and path smoothing, processes the initial path to obtain a feasible path (Lines 21–22).
**Algorithm 3:** Build Improved Heuristic Bi-RRT ($P_{init}$,$P_{goal}$)1: $P_{init}\leftarrow Current\_Root\;(\;)$; 2: $T_{a}\;(P_{init})$;$T_{b}\;(P_{goal})$; 3: $P_{judge1}\leftarrow P_{init}$;$P_{judge2}\leftarrow P_{goal}$; 4: **while** $Obstacle\_Collision\;(P_{judge1}\;,\;P_{judge2})$ **do** 5: $P_{rand}\leftarrow Heuristic\_Random\_Sampling\;(\;)$; 6: $P_{near}\leftarrow Heuristic\_Nearest\_Neighbor(P_{rand},T_{a})$; 7: $P_{new}\leftarrow Heuristic\_Extend\;(\;P_{near},\;P_{rand})$; 8: **if** $Collision\_Free\;(\;P_{near},\;P_{new})$ **then** 9:  $T_{a}.Add(P_{new}),\;T_{a}.Add(P_{near},P_{new})$ 10:  $P_{judge1}\leftarrow P_{new}$; 11:  $P_{nearest}\leftarrow Nearest\_Neighbor\;(P_{new},T_{b})$; 12:  $P_{judge2}\leftarrow P_{nearest}$; 13:  **if** $Collision\_Free\;(P_{judge1},P_{judge2})$ **then** 14:   Return T (Ta,Tb) 15:   break 16:  **else** $Swap\;(T_{a},T_{b})$ 17:  **end if** 18: **end if** 19: **end while** 20: $Path\leftarrow Get\_Path\;T\;(T_{a},T_{b})$; 21: Path←Heuristic_Reconnection (Path); 22: Trajectory←Smoothing (Path);

The improved heuristic Bi-RRT algorithm contains a set of heuristic methods to the benefit of path planning of the autonomous vehicle. The improvements in the connective mode of two random trees, node sampling, node selection, and node expansion based on the Bi-RRT framework are used to generate an initial path quickly. Additionally, then, path reorganization, including path reconnection and path smoothing, is employed to improve the quality of the initial path, making it suitable for tracking by the autonomous vehicle. The improved constraints containing the improved road environment and the improved vehicle constraint are conducive to generating a feasible path complying with the driver’s driving habit. In addition, a novel path coherence method is introduced to make the generated path smoothly connected when planning a dynamic obstacle avoidance path. Specific methods of the algorithm are described as follows in detail.

### 3.1. Connective Mode of Two Random Trees

**Figure 5:**
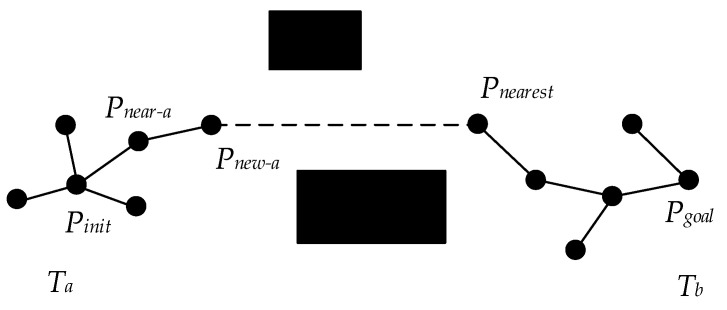
Obstacle-free direct connection of two random trees.

In order to further accelerate the running speed of the algorithm, the connective mode of two random trees can change from how the distance between two random trees is less than a certain distance threshold to the way of obstacle-free direct connection, as shown in Figure 5. After expanding the random tree $T_{a}$ to obtain a new tree node $P_{new-a}$, the nearest node $P_{nearest}$ on the random tree $T_{b}$ closest to the node $P_{new-a}$ is calculated, and whether the area between $P_{new-a}$ and $P_{nearest}$ is passable is checked. If there are no obstacles between $P_{new-a}$ and $P_{nearest}$, $P_{new-a}$ and $P_{nearest}$ are directly connected, and the initial path planning is complete. Otherwise, the algorithm continues to execute until the two random trees are connected successfully.

### 3.2. Heuristic Target Bias Sampling Method

The basic Bi-RRT usually adopts random searches in the global scope during the random sampling process, which will cause the generation of random points without guidance and too much unnecessary computation. As to this problem, a heuristic bias sampling strategy composed of a multiple-sampling method and a target bias method is adopted to make the random tree grow in a biased direction, enabling the initial state and the goal state to meet more effectively and faster. Equations (3) and (4) demonstrate how to calculate the random sampling state $P_{rand}$.

(3) $$P_{randm}=Heuristic\_Random\_State\;(\;)$$

(4) $$P_{rand}=\left\{\begin{array}{l} P_{randm}\;\;\;\;\;\;\;\;\;\;\;\;d_{\mathrm{ob}}\leq d_{\mathrm{threshold}}\\ P_{randm}+\chi \cdot \left(\frac{\vec{P_{randm}P_{t\arg et}}}{\left|\vec{P_{randm}P_{t\arg et}}\right|}\right)\;\;\;d_{\mathrm{ob}}>d_{\mathrm{threshold}}\; \end{array}\right.$$

where $P_{randm}$ is the random sampling state generated by the multiple-sampling method, $P_{t\arg et}$ is the target point defined in each sampling process, and χ is the biased step size. $d_{\mathrm{ob}}$ and $d_{\mathrm{threshold}}$ are the distance from the random point $P_{randm}$ to the obstacle and the distance threshold from the obstacle, respectively.

#### 3.2.1. Heuristic Random Sampling

With the knowledge of the initial and goal state, to make the sampling random point close to the target state, the Multiple_Random_State function generates several random points instead of one random point generated by the basic Bi-RRT algorithm in the free region using the Random_State function. The multiple-sampling point function does not include the probability of bias to the target, hence avoiding the local minimum problem. Additionally, then, the introduction of the Nearest_To_Target function is used to select the random state closer to the goal from several candidate sampling points. The random point out of these candidate sampling points closest to the target becomes the chosen random state $P_{randm}$, causing the Bi-RRT to search toward the target point. The number of random states to be selected was set to two after simulation results showed the best performance when the number of random states was limited to two.

#### 3.2.2. Heuristic Target Bias

After obtaining the chosen random state $P_{randm}$, the target bias method is introduced. It can make the random point expand a step size χ along the direction from it to the target state to generate the random sampling point further closer to the target state, resulting in making the random tree grow more directionally and improving computational efficiency. When $d_{\mathrm{ob}}$ is less than $d_{\mathrm{threshold}}$, if the generated random sampling point is still closer to the target state, it will make the newly generated tree node easy to collide with the obstacle, resulting in sampling failure and directly affecting the solution speed. Therefore, the target bias method introduces the distance threshold $d_{\mathrm{threshold}}$, which is the projection distance of the semi-major axis of the expanded safety ellipse on the x axis of the coordinate reference frame. When $d_{\mathrm{ob}}\leq d_{\mathrm{threshold}}$, that is, the chosen random state $P_{randm}$ is close to the obstacle, $P_{randm}$ is regarded as the generated random sampling point $P_{rand}$, when $d_{ob}>d_{\mathrm{threshold}}$, that is, the chosen random state $P_{randm}$ is far from the obstacle, $P_{rand}$ is generated from the target bias function. This strategy can maintain the balance between exploration and search speed.

Based on the basic Bi-RRT sampling function framework, the novel target bias sampling method changes the generation mode of the sampling point to make the generated sampling points more directional and effective, improving search efficiency and accelerating the algorithm’s convergence. Algorithm 4 outlines the working of the function Heuristic_Random_Sampling ( ). The improved heuristic Bi-RRT obtains candidate sampling points through the sampling function Random_State ( ) and selects the sampling point with the minimum distance cost as the current sampling point $P_{randm}$ (Lines 1–3). Then, according to the distance between the current sampling point $P_{randm}$ and the obstacle, the current sampling point $P_{randm}$ is processed by the heuristic target bias method to obtain the final sampling point $P_{rand}$ (Lines 4–7). This procedure is performed in each sampling process.
**Algorithm 4:** Function Heuristic_Random_Sampling ( )1: $P_{rand1}\leftarrow Random\_State\;(\;)$; 2: $P_{rand2}\leftarrow Random\_State\;(\;)$; 3: $P_{randm}\leftarrow Nearest\_To\_T\arg et\;(P_{rand1},P_{rand2})$; 4: **if** $d_{ob}\leq d_{threshold}$ **then** 5: $P_{rand}\leftarrow P_{randm}$; 6: **else** 7: $P_{rand}\leftarrow P_{randm}+\chi \cdot \left(\frac{\vec{P_{randm}P_{t\arg et}}}{\left|\vec{P_{randm}P_{t\arg et}}\right|}\right)\;$; 8: **end if**

### 3.3. Heuristic Parent Node Selection Method

**Figure 6:**
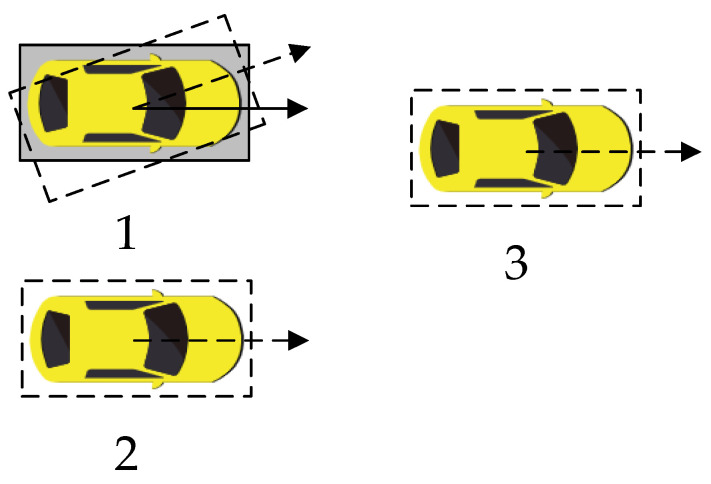
Different driving states.

In the basic Bi-RRT algorithm, the nearest tree node is measured by the Euclidean distance from the random point to the tree node, which may generate a polyline path with sharp included angles between connecting line segments of path nodes. Even if the polyline line path is smoothed, it cannot be successfully followed by vehicles. Because the vehicle has a minimum steering radius in actual motion, the Euclidean distance should not be the only factor to consider during each node selection process. As shown in Figure 6, the current vehicle state is a solid rectangle containing a yellow vehicle with three alternative driving states shown as dashed rectangles. In order to find the nearest driving state for the current vehicle, if the Euclidean distance is only considered, there is no doubt that the first driving state is the closest with a Euclidean distance of zero and the second driving state takes second place. However, the first and second driving states, also named in situ steering and lateral translation, are not possible for the vehicle due to the minimum steering radius. Hence, the third driving state is more reasonable.

**Figure 7:**
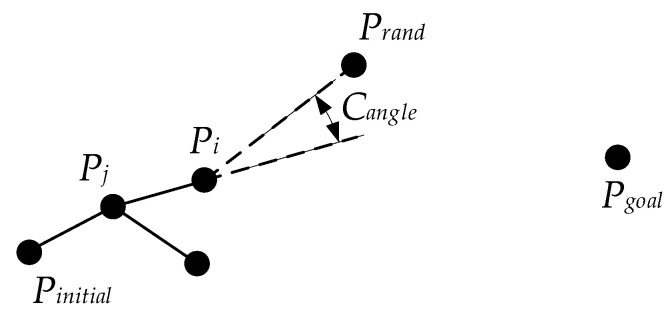
Calculation of the included angle.

Given the above analysis, the steering radius of the vehicle needs to be considered to choose the near tree node, namely the parent tree node. Therefore, a heuristic selection method of the parent tree node, namely a comprehensive measurement index considering the distance factor and angle factor, is introduced to make the generated path gentler and easily tracked by the vehicle. Furthermore, it can speed up the algorithm convergence and reduce the calculation time. Equations (5)–(11) show the calculation process when selecting the near tree node. The near tree node $P_{near}$ is determined as the tree node corresponding to the maximum comprehensive measurement index.

(5) $$C_{M}=\omega_{1}\cdot C_{dis\tan ce}^{1}+\omega_{2}\cdot C_{angle}^{1}$$

(6) $$C_{dis\tan ce}=\xi_{1}\cdot C_{dis\tan ce1}+\xi_{2}\cdot C_{dis\tan ce2}$$

(7) $$C_{dis\tan ce1}=\left|\vec{P_{tree}P_{rand}}\right|$$

(8) $$C_{dis\tan ce2}=\left|\vec{P_{tree}P_{goal}}\right|$$

(9) $$C_{angle}=\pi -\left(\cos^{-1}\left(\frac{\vec{P_{i}P_{j}}\cdot \vec{P_{i}P_{rand}}}{\left|\vec{P_{i}P_{j}}\right|\cdot \left|\vec{P_{i}P_{rand}}\right|}\right)\right)$$

(10) $$C_{dis\tan ce}^{1}=\frac{C_{dis\tan ce\_\max}-C_{dis\tan ce}}{C_{dis\tan ce\_\max}}$$

(11) $$C_{angle}^{1}=\frac{C_{angle\_\max}-C_{angle}}{C_{angle\_\max}}$$

where $\omega_{1}$ and $\omega_{2}$ are weighted coefficients of the distance index $C_{dis\tan ce}^{1}$ and the angle index $C_{angle}^{1}$, respectively. $C_{dis\tan ce}^{1}$ and $C_{angle}^{1}$ are the normalized values of the distance $C_{dis\tan ce}$ and the angle $C_{angle}$, respectively. The diagrammatic presentation of the angle $C_{angle}$ is shown in Figure 7. $C_{dis\tan ce}$ is the weighted sum of the distance $C_{dis\tan ce1}$ and the distance $C_{dis\tan ce2}$, $\xi_{1}$ and $\xi_{2}$ are the weighted coefficients of $C_{dis\tan ce1}$ and $C_{dis\tan ce2}$, respectively. $C_{dis\tan ce1}$ represents the distance from each tree node to the random sampling node, and $C_{dis\tan ce2}$ represents the distance from each tree node to the target state. The introduce of the distance $C_{dis\tan ce2}$ can make the selected near tree node have a trend close to the target state to increase the computational efficient.

The transition from Euclidean distance to the comprehensive measurement index changes the growing characteristic of the random tree. The introduction of the comprehensive measurement index hinders the pure expansion of the random tree growth towards the configuration region. On the contrary, it drives the tree growth towards the direction associated with the target state and the gentle trend. As a result, it improves the running speed of the algorithm to some extent, and there is a natural trade-off between quick tree growth and better path quality.

### 3.4. Heuristic Node Extension Method

**Figure 8:**
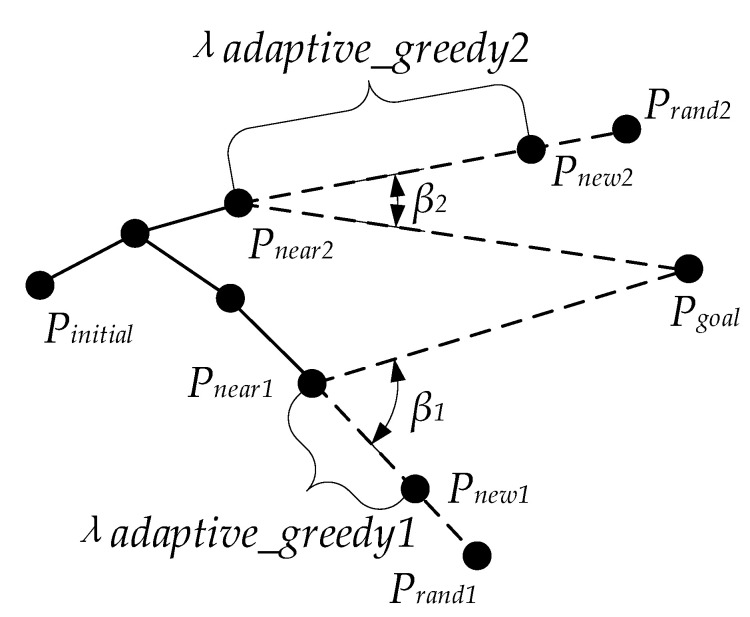
Adaptive greedy step size.

Expanding the near tree node to the random point usually adopts a fixed step size in the basic Bi-RRT. The large fixed step size tends to make the new node difficult to extend in the surrounding area of the obstacle, especially dense obstacle areas, resulting in extension failure and reducing the growth rate of the random tree. The small fixed step size could lead to a slow convergence speed of the algorithm. In other words, the fixed step size has the disadvantages of low flexibility and low security. In addition, the expansion direction of the tree node is along the vector direction from $P_{near}$ to $P_{rand}$. When utilizing the large step size to extend the parent tree node along the expansion direction of the tree node deviating from the target state, an invalid and unnecessary node could be generated, resulting in the low quality of the generated path, and affecting the running speed of the algorithm. Thus, to solve these two problems, a heuristic node extension method, namely the adaptive greedy step size, is introduced. ‘Adaptive’ is reflected in the pattern that the step size is dynamically and gradually changing rather than fixed, and ‘greedy’ is reflected in the pattern that the greater the extent of the node expansion direction approaching the direction of the target state, the larger the step size. Equations (12)–(16) show the calculation of the adaptive greedy step size, and its simple legend is shown in Figure 8.

(12) $$\lambda_{adaptive\_greedy}=\left\{\begin{array}{l} \lambda \;\;\;\;\;\;\;\;\;\;\;\;\;d_{near\_ob}<d_{threshold}\\ \left\{\begin{array}{l} (\eta_{\cos}+\sqrt{s})\cdot \lambda \;\;\;\;\;\beta <\frac{\pi }{2}\\ (1-\eta_{\cos}+\sqrt{s})\cdot \lambda \;\beta \geq \frac{\pi }{2}\; \end{array}\right.\;\;d_{near\_ob}\geq d_{threshold} \end{array}\right.$$

(13) $$\eta_{\cos}=\left|\frac{\vec{V_{unit\_r}}\cdot \vec{V_{unit\_t}}}{\left|\vec{V_{unit\_r}}\right|\cdot \left|\vec{V_{unit\_t}}\right|}\right|$$

(14) $$\beta =arc\cos\left(\frac{\vec{V_{unit\_r}}\cdot \vec{V_{unit\_t}}}{\left|\vec{V_{unit\_r}}\right|\cdot \left|\vec{V_{unit\_t}}\right|}\right)$$

(15) $$\vec{V_{unit\_t}}=\frac{\vec{P_{near}P_{t\arg et}}}{\left|\vec{P_{near}P_{t\arg et}}\right|}$$

(16) $$\vec{V_{unit\_r}}=\frac{\vec{P_{near}P_{rand}}}{\left|\vec{P_{near}P_{rand}}\right|}$$

where λ is the basic lower bound of the step size, $\vec{V_{unit\_t}}$ and $\vec{V_{unit\_r}}$ are unit vectors from $P_{near}$ to $P_{t\arg et}$ and from $P_{near}$ to $P_{rand}$, respectively. φ is the included angle between $\vec{V_{unit\_t}}$ and $\vec{V_{unit\_r}}$, $\eta_{\cos}$ is the cotangent of φ, s is a constant as a regulating coefficient, and $d_{near\_ob}$ is the distance from $P_{near}$ to the obstacle. Far away from the obstacle means $d_{nearest\_ob}\geq d_{threshold}$.

Compared with the traditional fixed step size, the adaptive greedy step size is no longer a constant. Its size is not only related to the distance from the obstacle but also to the included angle of the vector $\vec{P_{near}P_{rand}}$ towards the target state. In this way, when near an obstacle, a small step size can be used for the basic extension, making the search path more detailed and safer. When far from an obstacle, the step size can be greedily adjusted as the included angle changes, making the random tree adopt the larger step size to expand rapidly along the extension direction closer to the target state and reduce some blind expansions. As a result, the greedy mode of the step size accelerates the growth of the random tree and makes the growth of the path tend to approach the target state to a certain extent. It is exactly because of its dynamic adjustability that the adaptive greedy step size extension can often pass the obstacle detection when approaching the obstacle, unlike the fixed step size extension. Using the adaptive greedy step size can significantly improve the success rate of new node generation and accelerate overall search efficiency effectively.

### 3.5. Improved Constraints

Suppose a path can be successfully and effectively tracked by an autonomous vehicle. In that case, the path should not only meet the obstacle constraints but also comply with the driving characteristics of the actual driver, resulting in avoiding causing excessive tension and discomfort to the driver and passengers. The obstacle constraints include the road environment and the environmental constraint formed by the vehicle being regarded as an obstacle, namely, the vehicle constraint. Therefore, the road environment and the vehicle constraint are improved to obtain a feasible path meeting the driving habits of the actual driver.

#### 3.5.1. Improved Road Environment

**Figure 9:**
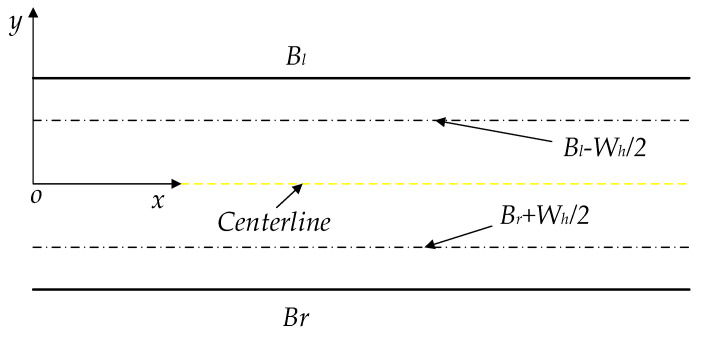
The improved road environment constraint.

In order to generate a practical path, the path nodes need to meet the constraints of road environments when planning. The road environment restricts the planned path more effectively by considering the vehicle width to avoid collision between the vehicle and the road edge. Thus, the newly extended nodes need to meet the requirements of the following Equation (17), and the schematic diagram of the road environment is shown in Figure 9.

(17) $$\left\{\begin{array}{l} P_{initial\_x}<T_{node\_x}<P_{goal\_x}\\ B_{r}+\frac{W_{h}}{2}<T_{node\_y}<B_{l}-\frac{W_{h}}{2} \end{array}\right.$$

where $P_{initial\_x}$ and $P_{goal\_x}$ are the x coordinates of the initial point and the goal point in the reference coordinate frame, respectively. $B_{l}$ and $B_{r}$ are the left and right boundaries of the road, and $W_{h}$ is the width of the host vehicle.

#### 3.5.2. Improved Vehicle Constraint

**Figure 10:**
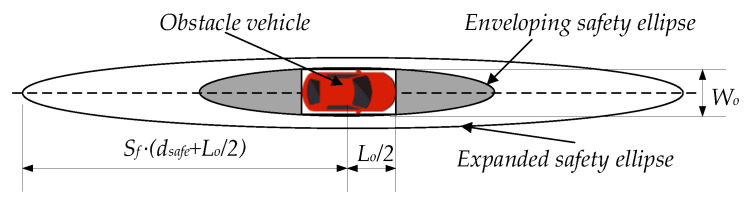
The improved vehicle constraint.

In order to express the constraint generated by the obstacle vehicle conveniently, a safety ellipse is introduced to envelop the obstacle vehicle. When considering the subjective comfort of the driver and passengers, a planned path needs to be able to avoid the obstacle vehicle in advance, that is, avoiding the generation of excessive path curvature when approaching the obstacle vehicle. In this case, the planned path can avoid causing discomfort to the driver and passengers and be easily tracked by the vehicle. Therefore, on the basis that half of the vehicle length is used as the semi-major axis of the safety ellipse, the advanced obstacle avoidance distance is added to the semi-major axis to ensure that the vehicle obstacle avoidance occurs at a long distance. Equation (18) expresses the condition that the newly expanded node needs to meet, and the schematic diagram of the vehicle constraint is shown in Figure 10.

(18) (x−x)obstacle2sf1⋅dsafe+Lo22+y−yobstacle2sf2⋅Wo2>1dsafe=v22⋅η⋅g

where (x,y) is the point on the connecting segment between the newly extended node $P_{new}$ and its parent node $P_{near}$, $\left(x_{ob},y_{ob}\right)$ is the position of the obstacle vehicle, $L_{o}$ and $W_{o}$ are the length and width of the obstacle vehicle, respectively. $s_{f1}$ and $s_{f2}$ are the expansion coefficients of the semi-major axis and the semi-minor axis of the safety ellipse, respectively, v is the speed of the host vehicle, η is the friction coefficient, g is the acceleration of gravity, and $d_{safe}$ is the advanced obstacle avoidance distance.

To sum up, during the process of the specific node extension, the improved constraints, including the improved road environment and the improved vehicle constraint, can make the planned path meet the actual driving requirement of the vehicle, namely, the path feasibility and the characteristics of avoiding the obstacle in advance.

### 3.6. Path Reorganization Method

Because the basic Bi-RRT algorithm adopts random sampling, the obtained path, also named the initial path, often has poor quality, mainly reflecting in containing too many unnecessary turn points and the discontinuity of path curvature. When tracking the initial path, the autonomous vehicle has to stop and change its driving direction so that it cannot drive smoothly and has unnecessary mechanical wear. Thus, a path reorganization method is needed to optimize the initial path, that is, to remove unnecessary turn points and smooth the path.

After obtaining an initial path by the function Get_Path T (Bi-T), as shown in Algorithm 2, the path reorganization method containing path node reconnection and path smoothing is used to process it. The path node reconnection is utilized to remove unnecessary turn nodes and insert path nodes to replace some necessary path nodes. As a result, it can make the included angles of the connecting line between path points meet the vehicle steering requirement, decreasing the control difficulty of the autonomous vehicle while reducing the path distance to the maximum extent. Additionally, then, the path obtained by the path node reconnection is a broken line composed of discrete path nodes; thus, it needs a further smoothing process to make the vehicle drive smoothly and steadily. Algorithm 5 describes the pseudocode of the path reorganization method.
**Algorithm 5:**  Function path_reorganization ( )
1: $Var\;S_{0},\;S_{1},\;S_{2}:\;path$ 2: $S_{0}(p_{n}\ldots ,p_{2},p_{1},p_{0})\leftarrow Get\_path\;T\;\left(Bi-T\right)$; 3: $p_{root}\leftarrow p_{0}$; $p_{parent}\leftarrow p_{1}$; $p_{current}\leftarrow p_{2}$; 4: $S_{1}\leftarrow (p_{root},p_{parent},p_{current})$; 5: $S_{2}\leftarrow (p_{3}\ldots p_{n})$; 6: **while** $p_{current}!=p_{n-1}$ **do** 7: **for** $each\;node\;p_{i}\in S_{2}$ **do** 8:  **if** $Collision\_Free\;(p_{current},p_{i})$ **then** 9:   $p_{forward}\leftarrow p_{i}$; 10:  **else** 11:  $p_{forward\_last}\leftarrow p_{i-1}$; 12:  **if** $(\pi -Angle\;(\vec{p_{current}p_{parent}},\;\vec{p_{current}p_{forward\_last}}))<\delta_{f}\leq \delta_{f\_\max}$ **then** 13:    $p_{root\_last}\leftarrow p_{root}$; $p_{root}\leftarrow p_{parent}$; $p_{parent}\leftarrow p_{current}$; $p_{current}\leftarrow q_{forward\_last}$; 14:    $S_{1}.Backward\_Add\_Node\;(p_{current})$; 15:  **else** 16:   **while** 1 **do** 17:    $p_{insert}\leftarrow Insert\_Node\;(p_{parent},p_{forward\_last})$; 18:    **if** $(\pi -Angle\;(\vec{p_{parent}p_{insert}},\;\vec{p_{parent}p_{root}}))<\delta_{f}\leq \delta_{f\_\max}$ and $(\pi -Angle\;(\vec{p_{insert}p_{parent}},\;\vec{p_{insert}p_{forward\_last}}))<\delta_{f}\leq \delta_{f\_\max}$ and $Collision\_Free\;(p_{parent},p_{insert})$ and $Collision\_Free\;(p_{insert},p_{forward\_last})$ **then** 19:      $p_{root\_last}\leftarrow p_{root}$; $p_{root}\leftarrow p_{parent}$; $p_{parent}\leftarrow p_{insert}$; $p_{current}\leftarrow p_{forward\_last}$; 20:      $S_{1}.Backward\_Add\_Node\;(p_{insert})$; 21:      $S_{1}.Backward\_Add\_Node\;(p_{\mathrm{current}})$; 22:     break 23:    **end if** 24:   **end while** 25:  **end if** 26: **end if** 27:  **end for** 28: **end while** 29: **if** $(\pi -Angle\;(\vec{p_{current}p_{parent}},\;\vec{p_{current}p_{n}}))<\delta_{f}\leq \delta_{f\_\max}$ **then** 30:  $S_{1}.Backward\_Add\_Node\;(p_{n})$; 31: **else** 32:  **while** 1 **do** 33:   $p_{insert}\leftarrow Insert\_Node\;(p_{root},p_{current})$; 34:  **if** $(\pi -Angle\;(\vec{p_{root}p_{root\_last}},\;\vec{p_{root}p_{insert}}))<\delta_{f}\leq \delta_{f\_\max}$  and $(\pi -Angle\;(\vec{p_{insert}p_{current}},\;\vec{p_{insert}p_{root}}))<\delta_{f}\leq \delta_{f\_\max}$ and $(\pi -Angle\;(\vec{p_{current}p_{insert}},\;\vec{p_{current}p_{n}}))<\delta_{f}\leq \delta_{f\_\max}$ and $Collision\_Free\;(p_{root},p_{insert})$ and $Collision\_Free\;(p_{insert},p_{current})$ **then** 35:    $S_{1}.Backward\_Delete\_Node\;(p_{end})$; 36:    $S_{1}.Backward\_Delete\_Node\;(p_{end-1})$; 37:    $S_{1}.Backward\_Add\_Node\;(p_{insert})$; 38:    $S_{1}.Backward\_Add\_Node\;(p_{current})$; 39:    $S_{1}.Backward\_Add\_Node\;(p_{n})$; 40:    break; 41:   **end if** 42:  **end while** 43: **end if** 44: $trajectory\leftarrow Cubic\_Bspline\;(S_{1})$; 45: Return trajectory


#### 3.6.1. Path Node Reconnection

A path obtained by the function Get_Path T (Bi-T) usually has poor connectivity due to the random attribute of the algorithm. Furthermore, there are many redundant turning segments in the path. As a result, it is often not continuously differentiable and infeasible. Thus, the path needs the path node reconnection to meet the prerequisite of path smoothing. Path node reconnection shown in lines 1–43 of Algorithm 5 is conducted to remove redundant path nodes and insert path nodes to replace some existing path nodes for obtaining a new path with maximum length reduction and no collision with obstacles. Moreover, it can ensure that the complementary angles of the included angles of line segments between path nodes are less than the steering angle of the front wheel. The function Get_Path T (Bi-T) is used to obtain a path node set $S_{0}$ of the initial path from the forward node of the goal node to the root node of the initial node (Line 2). The first path node is defined as the root node $p_{root}$, the second path node is defined as the parent node $p_{parent}$, and the third path node is defined as the current node $p_{current}$ (Line 3). A line segment connects the current node $p_{current}$ and the first subsequent path node from the set $S_{2}$. Additionally, then, connecting this current node $p_{current}$ and one of all subsequent path nodes from the set $S_{2}$ through a line segment successively in sequence is conducted until a collision occurs between the line segment and the obstacle (Lines 7–10). In this case, the path nodes between the line segment are removed, and the parent node of the path node that results in the collision with the obstacle is defined as the previous forward node $p_{forward\_last}$ (Line 11). Meanwhile, the complementary angle of the included angle between the vector $\vec{p_{current}p_{parent}}$ and the vector $\vec{p_{current}p_{forward\_last}}$ is calculated. When the complementary angle is less than the steering angle constraint $\delta_{f}$, $p_{root}$ is redefined as the previous root node $p_{root\_last}$, $p_{parent}$ is redefined as the new root node $p_{root}$, $p_{current}$ is redefined as the new parent node $p_{parent}$, and $p_{forward\_last}$ is redefined as the new current node $p_{current}$ (Lines 12–13). On the contrary, a path node $p_{insert}$ is inserted between the parent node $p_{parent}$ and the previous forward node $p_{forward\_last}$ to replace the current node $p_{current}$ and meet the conditions that the complementary angle of the included angle between the vector $\vec{p_{parent}p_{insert}}$ and the vector $\vec{p_{parent}p_{root}}$ and the complementary angle of the included angle between the vector $\vec{p_{insert}p_{parent}}$ and the vector $\vec{p_{insert}p_{forward\_last}}$ are less than the steering angle constraint $\delta_{f}$, and the line segment connecting the parent node $p_{parent}$ and the inserted node $p_{insert}$ and the line segment connecting the inserted node $q_{insert}$ and the previous forward node $p_{forward\_last}$ do not collide with the obstacle (Lines 17–18). After that, $p_{root}$ is redefined as the previous root node $p_{root\_last}$, $p_{parent}$ is redefined as the new root node $p_{root}$, $p_{insert}$ is redefined as the new parent node $p_{parent}$, and $p_{forward\_last}$ is redefined as the new current node $p_{current}$ (Line 19). The above operations are applied to the remaining path nodes in the set $S_{2}$ in sequence until the path node $p_{n-1}$ is found. Finally, suppose the complementary angle of the included angle between the line segment connecting the finally defined $p_{current}$ and the finally defined $p_{parent}$ and the line segment connecting the finally defined $p_{current}$ and $p_{n}$ is less than the steering angle constraint $\delta_{f}$. In that case, $p_{n}$ is added to the set $S_{1}$ (Lines 29–30). Otherwise, the final path node $p_{n}$ is added to the set $S_{1}$ after meeting the conditions that the complementary angle of the included angle between the line segment connecting the finally defined $p_{root}$ and the finally defined $p_{root\_last}$ and the line segment connecting the finally defined $p_{root}$ and the inserted node $p_{insert}$, the complementary angle of the included angle between the line segment connecting the inserted node $p_{insert}$ and the finally defined $p_{root}$ and the line segment connecting the inserted node $p_{insert}$ and the finally defined $p_{current}$, and the complementary angle of the included angle between the line segment connecting the finally defined $p_{current}$ and the inserted node $p_{insert}$ and the line segment connecting the finally defined $p_{current}$ and the final node $p_{n}$ are less than the steering angle constraint $\delta_{f}$, and the line segment connecting the finally defined $p_{root}$ and the inserted point $p_{insert}$ and the line segment connecting the inserted point $p_{insert}$ and the finally defined $p_{current}$ do not collide with the obstacle (Lines 31–43).

**Figure 11:**
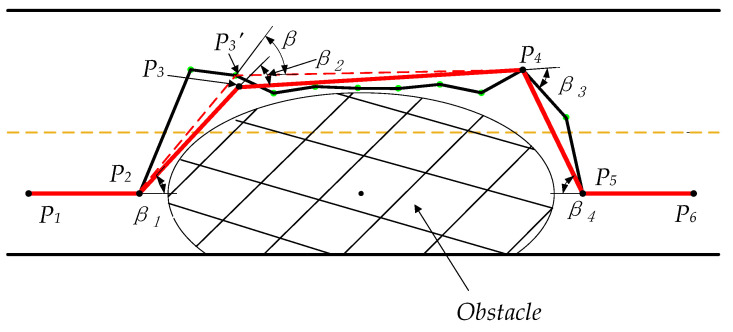
Path node reconnection processing.

The path node reconnection process is illustrated in Figure 11 specifically. The solid black line is the initial path obtained by the function Get_Path T (Bi-T). The connecting line segments between the black nodes $P_{1}$, $P_{2}$, $P_{3}$, $P_{4}$, $P_{5}$, and $P_{6}$, which are obtained by the process of the path node reconnection, constitute a path to be smoothed in the next step, which is represented as the red solid line. The connecting line segments between the black nodes $P_{2}$, $P_{3}$, $P_{4}$, and $P_{5}$ do not intersect with the obstacle, removing the redundant nodes between them, namely, the green path nodes. $P_{3}{}^{\prime }$, a path node from the initial path, can directly connect with $P_{2}$ and $P_{4}$. However, the complementary angle of the included angle between the line segment connecting $P_{3}{}^{\prime }$ and $P_{2}$, and the line segment connecting $P_{3}{}^{\prime }$ and $P_{4}$ is more than the steering angle constraint $\delta_{f}$. As a result, a node, namely, node $P_{3}$, is inserted between $P_{2}$ and $P_{4}$ based on the constraint $\delta_{f}$ to replace the node $P_{3}{}^{\prime }$ for obtaining a relatively gentle path with the maximum length reduction, namely the red path. Furthermore, the complementary angles of the included angles of line segments consisting of these black nodes are less than $\delta_{f}$, that is, $\beta_{1}$, $\beta_{2}$, $\beta_{3}$, and $\beta_{4}$ are less than $\delta_{f}$. Thus, the path node reconnection method can reduce the length of the initial path to the greatest extent and obtain a path convenient for further smoothing.

#### 3.6.2. Path Smoothing

After the path node reconnection process, a simplified path, the solid red line in Figure 11, is obtained, but the path contains some necessary turning nodes. As a result, the path is still not continuously differentiable such that it cannot be directly tracked by the vehicle. Thus, the obtained path should be further smoothed to make it be successfully tracked by the vehicle [40]. The cubic B-spline curve can move a control point to make the local modification without affecting the overall shape of the path and have a simple implementation and relatively low computational cost to ensure kinematic feasibility while avoiding an obstacle [41]. In addition, it is also often adopted to obtain a continuously differentiable cure [42]. Therefore, the cubic B-spline curve can be used to optimize the obtained path.

Supposing there are m+1 control points $P_{i}(i=0,1,\cdot \cdot \cdot ,m)$, the cubic B-spline curve is expressed as

(19) $$\left\{\begin{array}{l} P_{k,3}(t)=\sum_{i=0}^{3}P_{i+k}\cdot G_{i,3}(t)\;\;\;t\in [0,1)\\ k=0,1,\cdot \cdot \cdot m-3 \end{array}\right.$$

where the basic function $G^{i,3}(t)$ is defined as

(20) $$\left\{\begin{array}{l} G_{0,3}(t)=\frac{-t^{3}+3t^{2}-3t+1}{6}\\ G_{1,3}(t)=\frac{3t^{3}-6t^{2}+4}{6}\\ G_{2,3}(t)=\frac{-3t^{3}+3t^{2}+3t+1}{6}\\ G_{3,3}(t)=\frac{t^{3}}{6} \end{array}\right.$$

In order to make the cubic B-spline curve start from $P_{0}$, tangent to the vector $\vec{P_{0}P_{1}}$, end at $P_{m}$, and tangent to the vector $\vec{P_{m}P_{m-1}}$, the control points $P_{-1}$ and $P_{m+1}$ are added to meet the conditions $P_{-1}+P_{1}=2P_{0}$ and $P_{m-1}+P_{m+1}=2P_{m}$.

The path node organization method can make the generated path continuously differentiable while reducing path length to the maximum extent. Meanwhile, the generated path does not have unnecessary steering and meets the tracking requirement of the vehicle.

### 3.7. Path Coherence Method

Due to the randomness of the algorithm, the difference in the planned paths between two adjacent frames could easily cause the vehicle to shake during driving, If the difference is too large, it may even cause the vehicle to collide with an obstacle. Hence, to solve this path planning problem in dynamic environments, the interframe path coherence method is introduced to guarantee the smooth connection of planned paths between frames. The interframe path coherence method refers to the need to consider the information of the planned path of the previous frame when planning a path in the current frame. Its main idea is that at the beginning of each new planning cycle, the root node $P_{root\_newly}$ of the new planning cycle is the position whose distance from the root node in the trajectory planned in the previous planning cycle is equal to R. At the same time, a point along the tangent line of the newly generated root node is selected as the new search starting node $P_{initial\_newly}$. Equations (21) and (22) express the calculation of the root node $P_{root\_newly}$. Equation (23) shows how to calculate $P_{initial\_newly}$.

(21) $$\left\{\begin{array}{l} \left\{\begin{array}{l} P_{k,3}(t)=\sum_{i=0}^{3}P_{i+k}\cdot G_{i,3}(t)\;\;\;\;t\in [0,1)\\ k=0,1,\cdot \cdot \cdot m-3 \end{array}\right.\\ {\left(x-P_{root\_x}\right)}^{2}+{\left(y-P_{root\_y}\right)}^{2}=R^{2} \end{array}\right.$$

**Figure 12:**
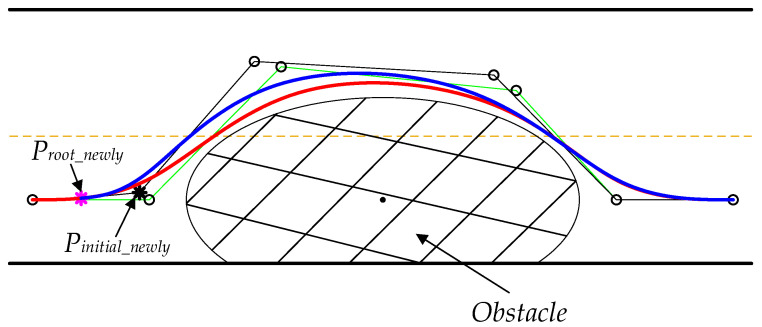
Path coherence illustration.

(22) R=ρ⋅v

(23) $$\left\{\begin{array}{l} \vartheta =\tan^{-1}(B_{slope})\\ P_{root\_newly}=P_{initial\_newly}+L_{skew}(\cos\vartheta ,\sin\vartheta ) \end{array}\right.$$

where $P_{root}$ is the root node of the previous planning cycle, R is the offset distance of the root node $P_{root}$, and ρ is the coefficient of proportionality. R is more than the preview distance of the vehicle to ensure that the preview point is still on the trajectory of the previous frame. As a result, there is no need to make more tracking control adjustments. $P_{root\_newly}$ is the right intersection point of the cubic B-spline curve and the designed circle. $B_{slope}$ is the slope of the cubic B-spline curve at the intersection point, ϑ is the included angle between the tangent line and the x axis at the intersection point, and $L_{skew}$ is the offset distance along the direction of the tangent line. $P_{root\_newly}$ and $P_{initial\_newly}$ are introduced to ensure that the trajectory generated in the current frame is tangent to the tangent line at the intersection point $P_{root\_newly}$ and passes through the intersection point $P_{root\_newly}$. Thereby, there are smooth connections in the interframe paths. As seen in Figure 12, the solid green line refers to the broken line formed by the path control points of the previous frame, and the solid black line is the broken line formed by the path control points of the current frame. The red curve represents the trajectory generated in the previous frame, the blue curve represents the trajectory generated in the current frame, and both curves are tangent to the vector $\vec{P_{root\_newly}P_{initial\_newly}}$ at the root node $P_{root\_newly}$. Hence, those two paths are smoothly connected at $P_{root\_newly}$.

The path coherence method can significantly eliminate the difference between interframe trajectories and make the planned trajectory smooth and continuous, resulting in maintaining the vehicle’s overall stability during driving.

## 4. Simulation Results and Analysis

### 4.1. Simulation Environment

**Figure 13:**
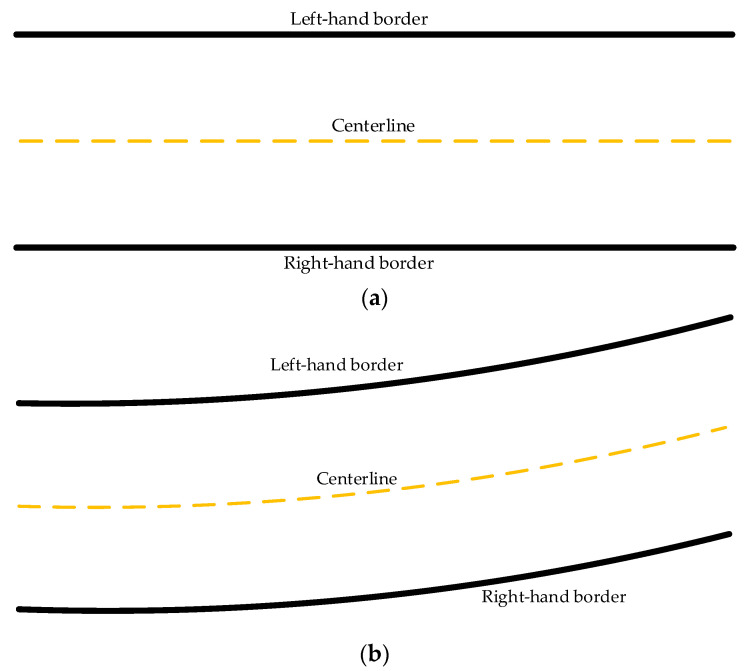
Road scenarios. (**a**) Straight road scenario. (**b**) Curved road scenario.

In order to verify the performance of the improved heuristic Bi-RRT algorithm, two typical road scenarios, including a straight road scenario and a curved road scenario, as shown in Figure 13a,b, are separately taken into account. Road information and other parameters in the whole simulation are shown in Table 1 and Table 2. The performance comparison of different RRT variants in the static straight road and curved road scenarios is conducted to verify the superiority of the improved heuristic Bi-RRT method. Meanwhile, interframe path planning and tracking are performed in dynamic scenarios to verify the effectiveness of the proposed algorithm. The simulations were performed on a PC with processor Intel I7 based on MATLAB R2019b and Carsim 2018. MATLAB R2019b is a mathematical software developed by Mathworks in Massachusetts, USA, and Carsim 2018 is a vehicle system simulation software developed by Mechanical Simulation Corporation in Michigan, USA.

### 4.2. Performance Measure of Path Planning

**Figure 14:**
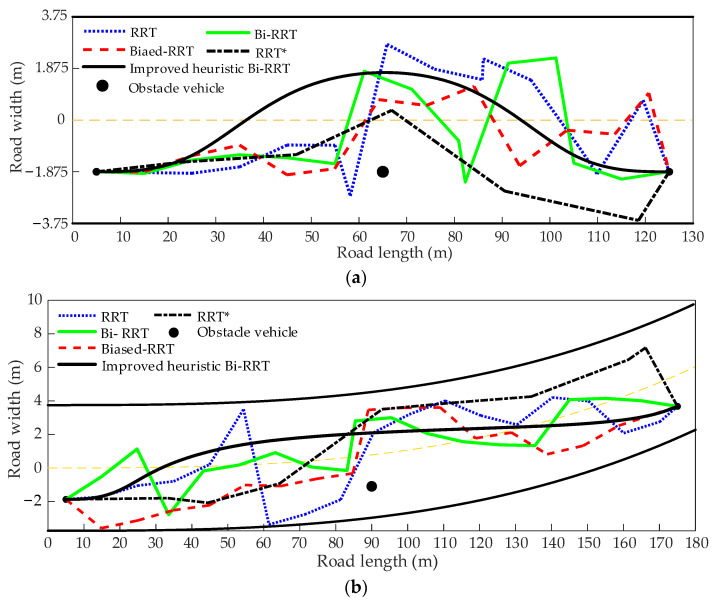
The simulation paths. (**a**) The planned paths in the straight road scenario. (**b**) The planned paths in the curved road scenario.

**Figure 15:**
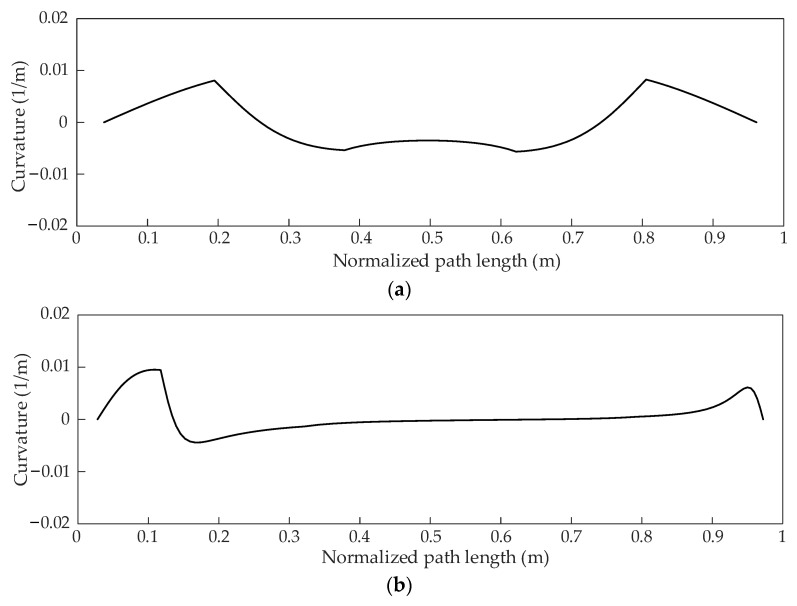
The simulation path curvatures. (**a**) The path curvature in straight road scenario. (**b**) The path curvature in curved road scenario.

The improved heuristic Bi-RRT algorithm is compared with the basic RRT, the biased RRT, the Bi-RRT, and the RRT* in the straight and curved road scenarios, and the results are shown in Figure 14a,b, respectively. The blue dotted line represents the path planned by the basic RRT, the red dashed line represents the path planned by the biased RRT, and the black dash-dotted line represents the path planned by the RRT*. The solid green and black lines represent the paths planned by the Bi-RRT and the improved heuristic Bi-RRT, respectively. It can be seen in Figure 14a,b that the paths generated by the basic RRT, the Bi-RRT, and the biased RRT contain a large number of corners, and there are frequent large curvature changes. In contrast, the path generated by the RRT* is relatively gentle, but it is still a broken line, which does not meet the steering requirement of the vehicle. However, the path generated by the improved heuristic Bi-RRT is a continuous and smooth curve, and the path curvature is also continuous, as shown in Figure 15a,b, respectively, resulting in the convenience being well followed by the vehicle. Observing Figure 14a,b, all paths planned by the basic RRT, the biased RRT, the Bi-RRT, the RRT*, and the improved heuristic Bi-RRT can successfully avoid the obstacle vehicle. However, there are differences in obstacle avoidance modes. The paths planned by the basic RRT, the biased RRT, the Bi-RRT, and the RRT* always start emergency obstacle avoidance when approaching the obstacle vehicle. The path planned by the proposed algorithm can start obstacle avoidance in advance by a safe distance from the obstacle vehicle, ensuring safe driving, conforming to the driver’s behavior habit, and not bringing excessive tension to passengers. The proposed algorithm can realize obstacle avoidance in advance because the obstacle avoidance distance is embedded when constructing the obstacle vehicle constraint.

A set of benchmarking parameters is defined to objectively compare the performance of the improved heuristic Bi-RRT and some RRT variants. The number of nodes on the mature random tree is denoted by “tree nodes.” The length of the generated path is denoted by “path length.” The searching time of path planning is denoted by “time.” In addition, the number of segments comprising the generated path is donated by “path segments”; in particular, the path segments of the improved heuristic RRT refer to the number of segments of the path after being processed by path reconnection [30,43,44].

Thirty independent simulation experiments were implemented on each algorithm to offset the random deviation of a single experiment, and the results are shown in Table 3 and Table 4. The average path length generated by the RRT* algorithm is much lower than those of the basic RRT, the biased RRT, and the Bi-RRT because the RRT * embedded with the reconnection mechanism can approach the approximate shortest path. However, the average path length generated by the improved heuristic Bi-RRT algorithm is smaller than that of the RRT* algorithm, and the average tree nodes and the average path segments of the proposed algorithm are also minimal compared with those of the other four algorithms. The decrease in path length and number of path segments is mainly due to the introduction of the path node reconnection method. The relatively small number of path segments can reduce the control difficulty of the vehicle and make the further smoothed path not contain unnecessary turns. In terms of the average planned time, the performance of the improved heuristic Bi-RRT algorithm is optimal compared with those of the other four algorithms, and especially in the straight road scenario, the planning time of the improved heuristic Bi-RRT algorithm can reduce significantly.

As a result of the performance measure, the improved heuristic Bi-RRT algorithm shows superior performance regarding path quality and planning time compared with the basic RRT, the biased RRT, the Bi-RRT, and the RRT*.

### 4.3. Path Coherence Validation

The dynamic environment can usually be treated as a static environment in a dynamic path planning process. That is, the re-planning of paths needs to be conducted in each frame environment region obtained by the perception module. Thus, the running speed of the path planning algorithm should not be only considered, but also the interframe connection of the planned paths should be concerned. In order to further verify the superior performance of the improved heuristic Bi-RRT algorithm, the planning effect of the corresponding algorithm at a specific frame is described in detail.

In order to express the coherence between the front and back frame paths, the real-time re-planning is carried out in two dynamic driving scenarios, where the host vehicle and the obstacle vehicle move in opposite and the same directions, respectively. The red triangle represents the position of the obstacle vehicle in the current frame, the yellow arrow represents the driving direction of the obstacle vehicle, and the green solid point and the black solid point represent the start point and end point of the current frame, respectively.

**Figure 16:**
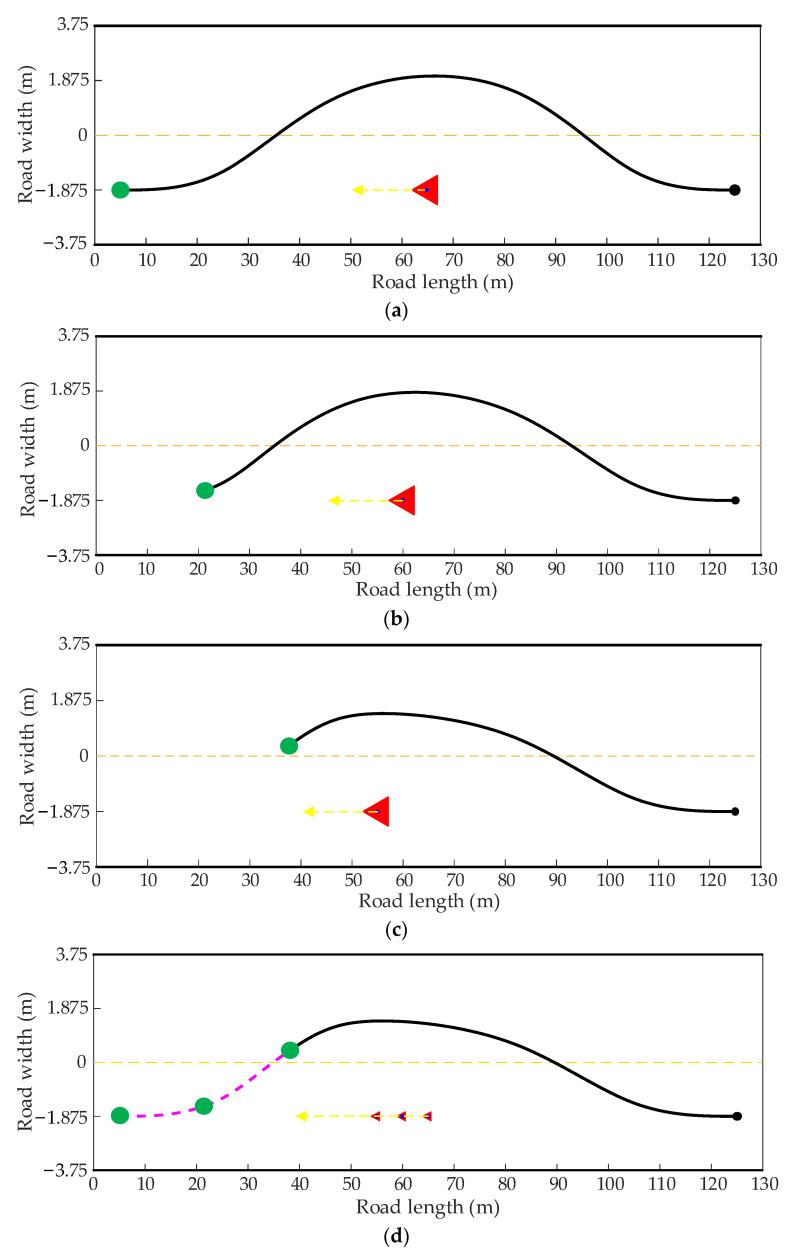

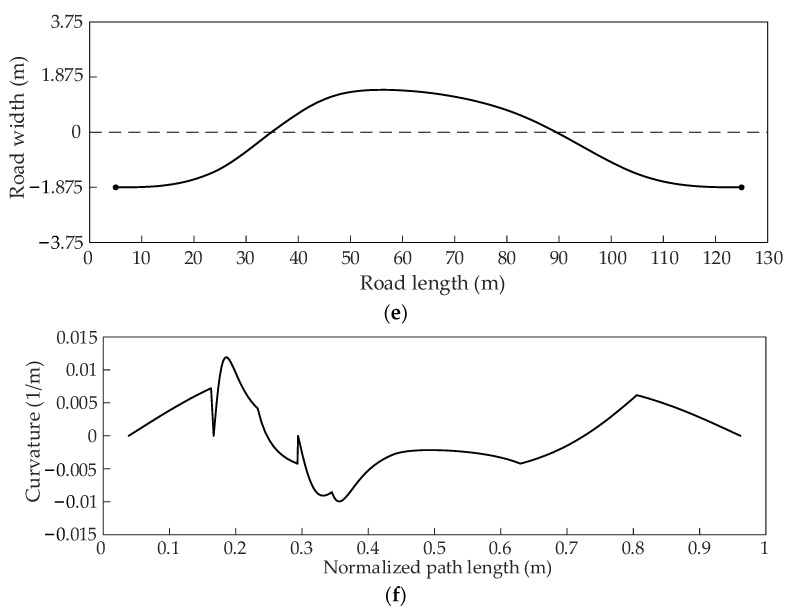
The path planning results in a dynamic scenario with driving in opposite directions. (**a**) The planned path in the first frame. (**b**) The planned path in the second frame. (**c**) The planned path in the third frame. (**d**) The path coherence process of three frames. (**e**) The coherence path of three frames. (**f**) The curvature of the coherence path.

Figure 16a–c show the dynamic path planning process of the improved heuristic Bi-RRT algorithm for three consecutive frames in the dynamic scenario with driving in opposite directions. Observing Figure 16a–c, the paths obtained in the first, second, and third frames are smooth and successfully bypass the moving obstacle vehicle. It can be seen from Figure 16d that the paths, magenta lines, obtained in the first frame, the second frame, and the third frame can be smoothly connected to form a smooth coherence path, as shown in Figure 16e. Moreover, the curvature of the coherence path is continuous and varies in a small range, which is convenient for it to be tracked by the host vehicle, as shown in Figure 16f.

**Figure 17:**
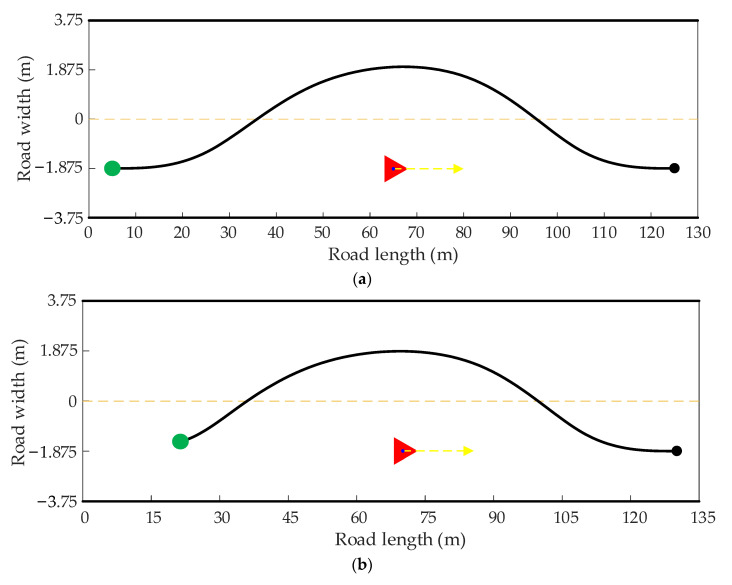

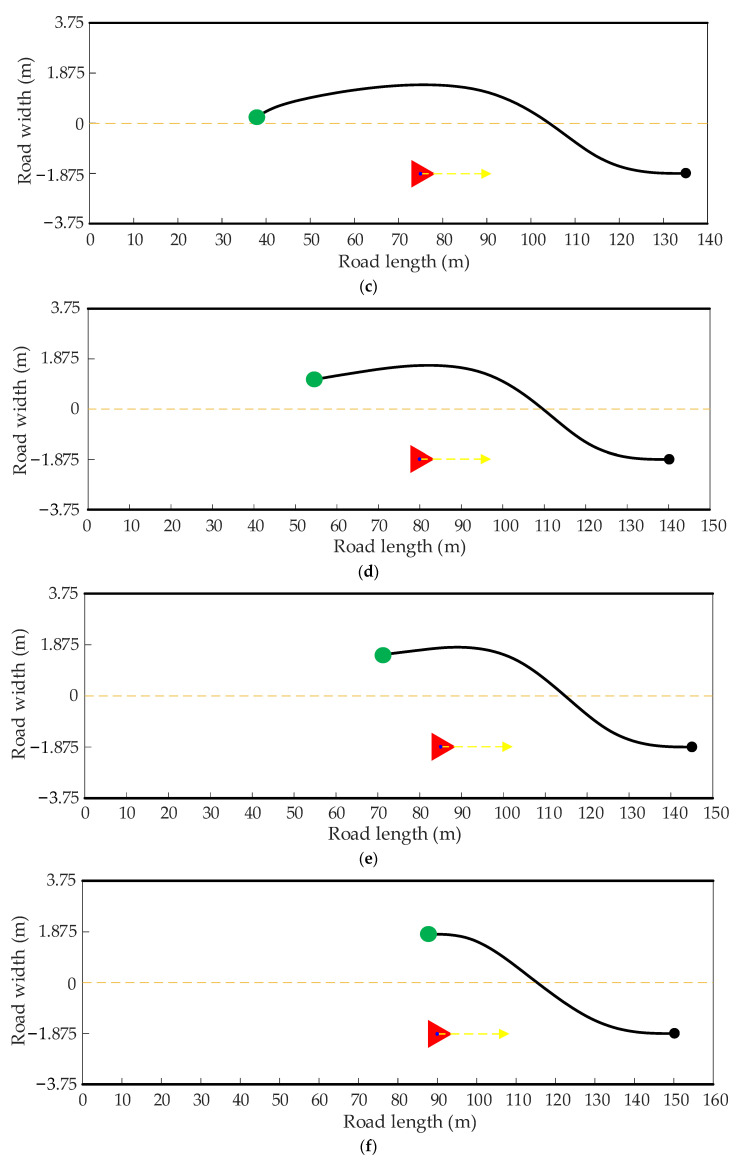

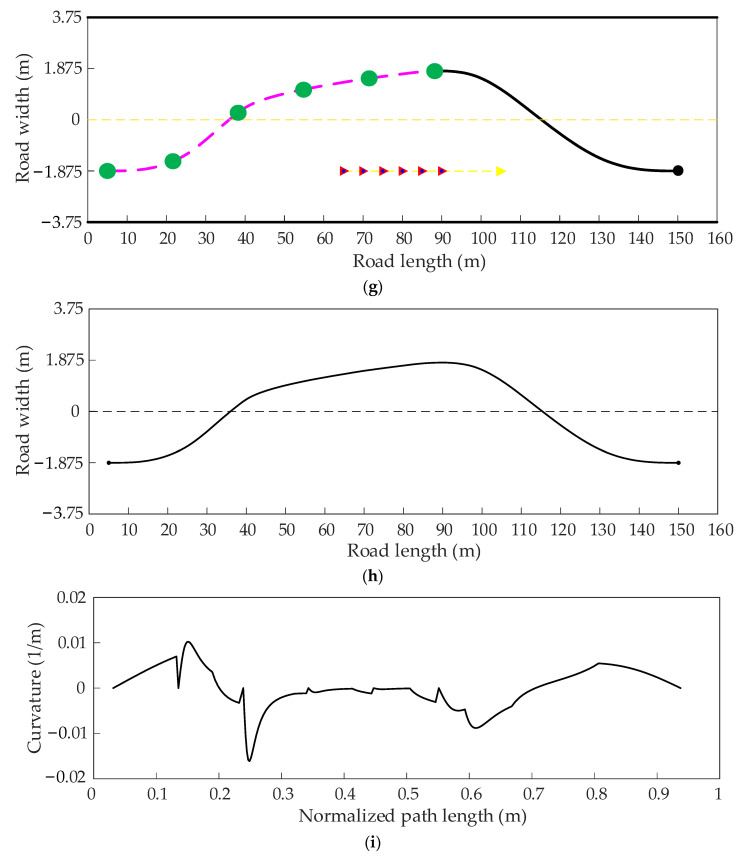
The path planning results in a dynamic scenario with driving in the same direction. (**a**) The planned path in the first frame. (**b**) The planned path in the second frame. (**c**) The planned path in the third frame. (**d**) The planned path in the fourth frame. (**e**) The planned path in the fifth frame. (**f**) The planned path in the sixth frame. (**g**) The path coherence process of six frames. (**h**) The coherence path of six frames. (**i**) The curvature of the coherence path.

Figure 17a–f show the dynamic path planning process of the improved heuristic Bi-RRT algorithm for six consecutive frames in the dynamic scenario with driving in the same direction. It can be seen from Figure 17a–f that the paths obtained in the first, second, third, fourth, fifth, and sixth frames are smooth and also successfully bypass the moving obstacle vehicle. As seen in Figure 17g, the paths, magenta lines, obtained in the first frame, the second frame, the third frame, the fourth frame, the fifth frame, and the sixth frame can be smoothly connected to generate a smooth coherence path, as shown in Figure 17h. In addition, the curvature of the obtained coherence path is continuous and within the range of 0.02 1/m, as shown in Figure 17i, resulting in the condition that the host vehicle can easily track the coherence path.

For further verifying the tracking performance of the obtained coherence paths shown in Figure 16e and Figure 17h, the path following experiments can be carried out in the Carsim simulation platform. The appropriate vehicle and driver models are selected to simulate the driving state of the real vehicle. The solid black and red dashed lines represent the target and followed paths, respectively.

**Figure 18:**
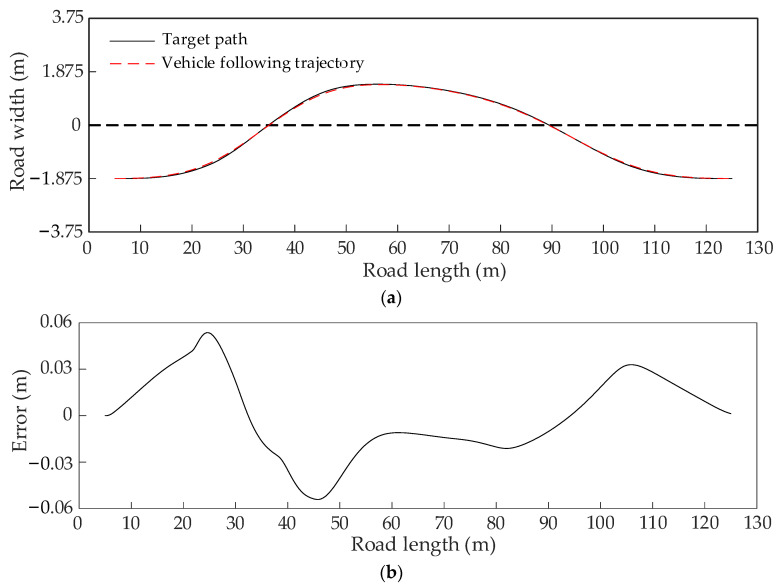

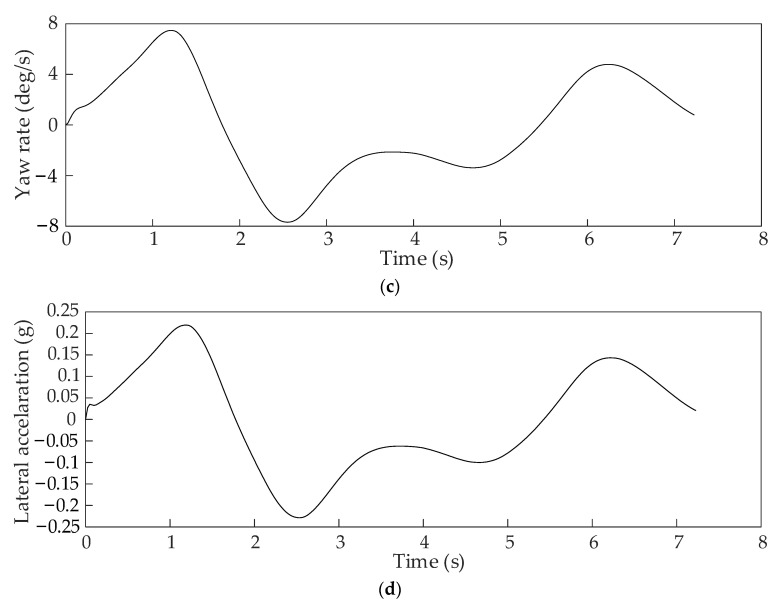
The path tracking results of the coherence path in a dynamic scenario with driving in opposite directions. (**a**) The path following result. (**b**) The path following error. (**c**) The yaw velocity. (**d**) The lateral acceleration.

The path following experiment in the dynamic scenario with driving in opposite directions is conducted. The result shown in Figure 18a represents the target path and the followed path. The following error between the followed path and the target path is relatively small and within the range of 0.06 m, as shown in Figure 18b, resulting in making the following effect acceptable. The yaw rate is within the range of 8 deg/s, as shown in Figure 18c, and the lateral acceleration is within the range of 0.25 g and less than the usual value of 0.8 g of the maximum lateral acceleration of steady driving, as shown in Figure 18d. These two indicators prove that the dynamic performance of the host vehicle is stable in the simulation experiment. Based on these facts, the obtained coherence path is satisfactory and effective.

**Figure 19:**
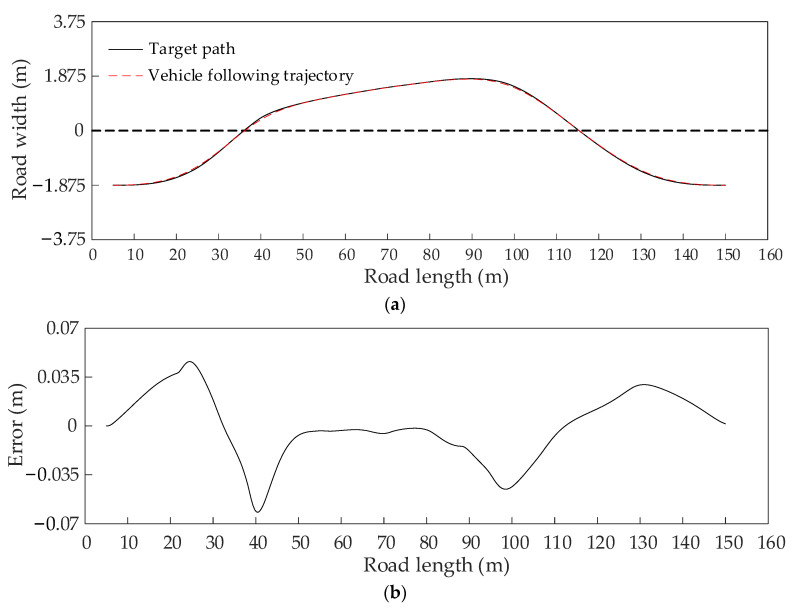

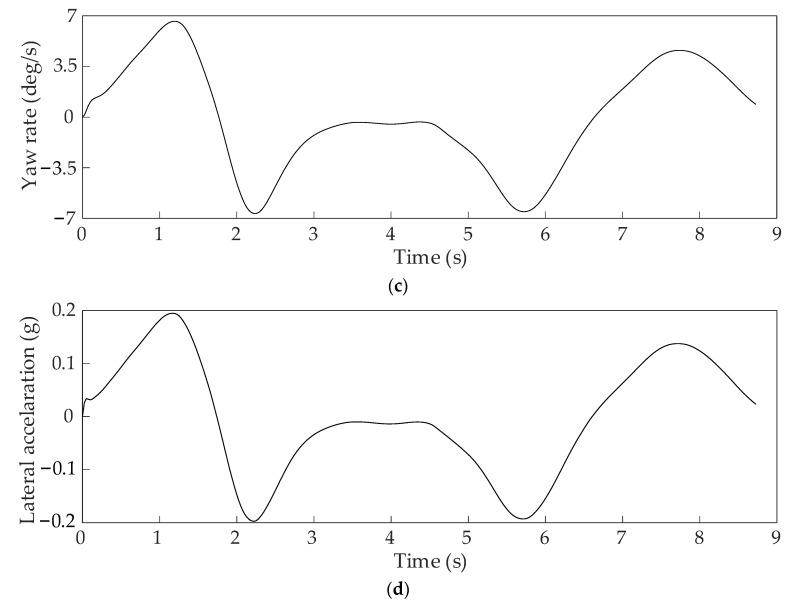
The path tracking results of the coherence path in a dynamic scenario with driving in the same direction. (**a**) The path following result. (**b**) The path following error. (**c**) The yaw velocity. (**d**) The lateral acceleration.

The path following experiment in the dynamic scenario with driving in the same direction is conducted. The target path and the followed path are shown in Figure 19a. The following error between the followed path and the target path is within the range of 0.07 m, as shown in Figure 19b, and is relatively small, which shows that the coherence path has a satisfactory tracking effect. The yaw rate and the later acceleration of the host vehicle when following the trajectory are shown in Figure 19c,d, respectively. The yaw rate is within the range of 7 deg/s, and the lateral acceleration is within the range of 0.2 g and less than the usual value of 0.8 g of the maximum lateral acceleration of steady driving, which shows the excellent dynamic performance of the host vehicle in the simulation process. Thus, the obtained coherence path is feasible and acceptable.

It can be seen from the test results that the algorithm proposed in this article can plan a path with smooth transition connections and continuous curvature. Furthermore, it is applied successfully in the dynamic driving scenarios of opposite driving and traveling in the same direction, which are the most common in vehicle driving.

## 5. Discussion

The path planning of the vehicle in dynamic scenarios often pays attention not only to the quality of each frame path but also to the difference in paths between frames. In addition, the Bi-RRT algorithm, a variant of the basic RRT algorithm, is often used for path planning because of its probability completeness and rapidity. However, its planned path is not differentiable and relatively poor in length. Based on those facts, some improved heuristic methods are introduced to the Bi-RRT algorithm to make it suitable for the dynamic path planning of the vehicle. The multi-sampling method biased towards the target state makes the growth of the random tree directional and reasonable. The adaptive greedy step size considering the target direction can increase the success rate of node expansion and make the newly extended node tend to the target state direction to a certain extent. The two random trees are directly interconnected when there are no obstacles between them, as a result, which further reduces the running time of the algorithm. Changing from only considering the Euclidean distance to considering the distance from the target state and the included angles between the connection lines of tree nodes, the parent node selection method improves the running speed of the algorithm to a certain extent while making the generated path tend to be gentle. The path reorganization method, including path node reconnection and path smoothing, can remove necessary turning points, significantly reduce the path length, and plan a path with continuous curvature. As for the problem of path connection between frames, the path coherence method can make paths between different frames smoothly connect to form a differentiable path. By way of the simulation experiment study, the improved heuristic Bi-RRT algorithm has a real-time performance, especially in a straight road scenario, and guarantees the shortest path while obeying the road constraints and the vehicle constraint and considering the driving habit of the driver. On the contrary, the vehicle constraint and the driver’s driving habits are not considered when applying the basic Bi-RRT algorithm.

However, it must be admitted that the running time may not be fast enough when the proposed algorithm is applied in a high-speed and dynamic curved road scene, which may be due to a large number of numerical calculations based on graphic geometry in obstacle detection. In future research, obstacle detection of gray value comparison and parallel computing are introduced to further reduce the proposed algorithm’s running time to meet the path planning requirement in a high-speed curved road scenario. After the preparation of the perception module, changing from the numerical calculation mode of graphics geometry to the comparison of gray values mode on binarized images, the obstacle detection can greatly reduce the calculation amount of the algorithm, thus speeding up the search speed of the algorithm. On this basis, the improved heuristic Bi-RRT algorithm can be applied to more complex driving scenarios, such as unstructured road scenes with a mixture of static and moving obstacles with grooves, to explore its adaptability. Furthermore, compared with other related algorithms, finding out the algorithm’s shortcomings and the scenes where the algorithm cannot be applied are made to improve the algorithm and enable it to be applied to more driving scenes.

## 6. Conclusions

This paper is concerned with the path planning of autonomous vehicles in a dynamic environment. An improved heuristic Bi-RRT algorithm has been proposed and tested. The proposed algorithm can solve the path query problem of the basic Bi-RRT algorithm and the interconnection problem of paths between various frames in a dynamic scenario to obtain a smooth and asymptotically optimal path with continuous curvature with high efficiency and accuracy. The proposed path planning algorithm consists of the obstacle-free direct connection of two trees, the heuristic target bias sampling, the heuristic parent node selection, the heuristic node extension, the improved constraints, path reorganization, and path coherence. The obstacle-free direct connection mode can further accelerate the interconnection of the two random trees. The heuristic target bias sampling can reduce blind sampling, and the heuristic node extension can decrease invalid expansion, thereby accelerating the running speed and improving the searching efficiency of the algorithm. The heuristic parent node selection speeds up the algorithm’s calculation and improves path quality to some extent. The improved environmental constraint and the improved vehicle constraint integrated with the advanced obstacle avoidance distance are considered together to make the vehicle avoid the obstacle in advance and accurately and make the vehicle drive safely. Path reorganization aims to post-process the initial path to obtain a reasonable and differentiable path with the approximate optimal length, which can be tracked by the vehicle smoothly and successfully. In addition, path coherence solves the problem of the smooth connection of paths between different frames, enabling the vehicle to run smoothly and steadily at the connection point. Through the simulation experiments, the improved heuristic Bi-RRT algorithm can generate the smoothest path and takes the shortest time compared with the other four algorithms. As a result, it is an effective local path planning algorithm for the autonomous vehicle and has practical value in the application of the wheeled robot.

In future works, the research focuses on increasing the solution speed, further reducing the calculation time, especially in the curved road scenario. The proposed algorithm will be applied in more complex driving scenarios, such as the parking scene and the drift scene with moving obstacles, to test its adaptiveness. Moreover, after the preparation of the test platform of the autonomous vehicle, an on-site experiment is conducted to test its effectiveness in practical applications.

## Figures and Tables

**Table 1:** Road parameters.

Road Type	Transverse Length (m)	Lane Width (m)	Initial Point	Target Point	Obstacle Point
Straight	130	3.75	5, −1.875	125, −1.875	65, −1.875
Curve	180	3.75	5, −1.874	175, 3.676	90, −1.095

**Table 2:** Simulation parameters.

Parameter	Value	Parameter	Value
Obstacle vehicle width $W_{o}$ (m)	1.8	Expansion coefficient (straight) $s_{f1}$	$\sqrt{2}$
Obstacle vehicle length $L_{o}$ (m)	4.8	Expansion coefficient (straight) $s_{f2}$	$\sqrt{3}$
Biased step size χ (m)	3	Expansion coefficient (curve) $s_{f1}$	$\sqrt{3}$
Step size λ (m)	10	Expansion coefficient (curve) $s_{f2}$	$\sqrt{2}$
Regulating coefficient s	1.5	Weighted coefficient $\omega_{1}$	0.4
Host vehicle width $W_{h}$ (m)	1.8	Weighted coefficient $\omega_{2}$	0.6
Host vehicle speed v (km/h)	60	Weighted coefficient $\xi_{1}$	0.7
Friction coefficient η	0.8	Weighted coefficient $\xi_{2}$	0.3
Gravity acceleration g (m/s2)	9.8	Proportionality coefficient ρ	0.6
Constraint angle $\delta_{f}$ (°)	30		

**Table 3:** Performance measures in the straight road scenario.

Algorithm	RRT	Biased RRT	Bi-RRT	RRT*	Improved Heuristic Bi-RRT
Average tree nodes	38.733	28.567	17.667	52.967	6.033
Average path segments	14.267	13.433	13.367	6.233	3.000
Average path length (m)	123.977	122.176	122.781	120.961	120.299
Average time (s)	0.026	0.022	0.020	0.043	0.010

**Table 4:** Performance measures in the curve scenario.

Algorithm	RRT	Biased RRT	Bi-RRT	RRT*	Improved Heuristic Bi-RRT
Average tree nodes	41.500	30.733	23.067	39.067	20.500
Average path segments	18.333	18.233	18.267	8.267	3.000
Average path length (m)	173.834	172.414	172.899	170.848	170.152
Average time (s)	3.363	2.432	0.732	12.757	0.626

## Data Availability

Data is contained within the article as figures and tables.
